# Antenatal dexamethasone treatment transiently alters diastolic function in the mouse fetal heart

**DOI:** 10.1530/JOE-18-0666

**Published:** 2019-04-23

**Authors:** E J Agnew, A Garcia-Burgos, R V Richardson, H Manos, A J W Thomson, K Sooy, G Just, N Z M Homer, C M Moran, P J Brunton, G A Gray, K E Chapman

**Affiliations:** 1 Centre for Cardiovascular Science, The University of Edinburgh, The Queen’s Medical Research Institute, Edinburgh, UK; 2 Mass Spectrometry Core, Edinburgh Clinical Research Facility, Centre for Cardiovascular Science, The University of Edinburgh, The Queen’s Medical Research Institute, Edinburgh, UK; 3 Centre for Discovery Brain Sciences, The University of Edinburgh, Hugh Robson Building, George Square, Edinburgh, UK

**Keywords:** cardiovascular, corticosteroids, embryo, glucocorticoid receptor, steroids

## Abstract

Endogenous glucocorticoid action is important in the structural and functional maturation of the fetal heart. In fetal mice, although glucocorticoid concentrations are extremely low before E14.5, glucocorticoid receptor (GR) is expressed in the heart from E10.5. To investigate whether activation of cardiac GR prior to E14.5 induces precocious fetal heart maturation, we administered dexamethasone in the drinking water of pregnant dams from E12.5 to E15.5. To test the direct effects of glucocorticoids upon the cardiovascular system we used SMGRKO mice, with *Sm22-Cre*-mediated disruption of GR in cardiomyocytes and vascular smooth muscle. Contrary to expectations, echocardiography showed no advancement of functional maturation of the fetal heart. Moreover, litter size was decreased 2 days following cessation of antenatal glucocorticoid exposure, irrespective of fetal genotype. The myocardial performance index and E/A wave ratio, markers of fetal heart maturation, were not significantly affected by dexamethasone treatment in either genotype. Dexamethasone treatment transiently decreased the myocardial deceleration index (MDI; a marker of diastolic function), in control fetuses at E15.5, with recovery by E17.5, 2 days after cessation of treatment. MDI was lower in SMGRKO than in control fetuses and was unaffected by dexamethasone. The transient decrease in MDI was associated with repression of cardiac GR in control fetuses following dexamethasone treatment. Measurement of glucocorticoid levels in fetal tissue and hypothalamic corticotropin-releasing hormone (*Crh*) mRNA levels suggest complex and differential effects of dexamethasone treatment upon the hypothalamic–pituitary–adrenal axis between genotypes. These data suggest potentially detrimental and direct effects of antenatal glucocorticoid treatment upon fetal heart function.

## Introduction

Levels of glucocorticoid hormones rise markedly shortly prior to birth to promote fetal organ and tissue maturation in preparation for birth and subsequent postnatal life (Fowden *et al.* 1998). This is mimicked with the administration of potent synthetic glucocorticoids (betamethasone or dexamethasone) to pregnant women at risk of preterm delivery, in order to mature fetal organs, thereby reducing neonatal morbidity and mortality should preterm birth ensue (Roberts *et al.* 2017, Agnew *et al.* 2018). A great deal is known about the maturational effects that endogenous as well as exogenous glucocorticoids have on the lungs (Bird *et al.* 2015, Roberts *et al.* 2017). However, the role of glucocorticoids in the maturation of other fetal organs has not been well characterised and concerns have been raised that non-optimal antental glucocorticoid therapy might increase adverse outcomes, including perinatal death (reviewed in Kemp *et al.* 2018). The effects of endogenous glucocorticoids upon fetal heart maturation were, until recently, unclear. We demonstrated an important role for endogenous glucocorticoids, acting via the glucocorticoid receptor (GR), in fetal heart maturation. At embryonic day (E) 17.5, global GR-knockout mice show impaired heart structure and function and fail to show the normal maturational changes in late gestation cardiac expression of genes involved in contractile function, calcium handling and energy metabolism (Rog-Zielinska *et al.* 2013). Tissue-specific knock-out of GR by *smooth muscle protein 22* (*SM22*)-*Cre*-driven recombination showed that most of these maturational effects are attributable to direct actions of GR within cardiomyocytes and/or vascular smooth muscle cells (VSMCs) (Rog-Zielinska *et al.* 2013). Expression profiling and *in silico* transcription factor analysis in sheep heart suggest that endogenous glucocorticoids similarly promote the metabolic transitions that occur close to birth in large animals (Richards *et al.* 2015). In contrast, although a number of animal studies have investigated the effects of fetal exposure to exogenous glucocorticoids upon subsequent heart structure and function in adulthood (termed ‘fetal programming’; Rog-Zielinska *et al.* 2014, Agnew *et al.* 2018), few have examined the impact of exogenous glucocorticoid administration upon the heart *in utero*, likely to underpin the long-term effects. Studies in fetal sheep hearts have shown that late gestation maternal hypercortisolaemia impacts the myocyte transcriptome, with some changes indicative of precocious maturation, but others not (Richards *et al.* 2014). However, whether these effects are mediated by alterations in maternal or placental physiology or reflect direct actions upon fetal cardiomyocytes is unknown. A clear consensus regarding the direct impact of exogenous glucocorticoid upon the fetal heart is yet to emerge.

Here, we hypothesised that early activation of GR would advance fetal heart maturation and that this would be via direct activation of GR in fetal cardiomyocytes. We tested our hypothesis by administering antenatal dexamethasone in mice, from E12.5 to E15.5 (prior to the natural late gestation peak in endogenous glucocorticoid levels at E16.5 to E17.5). To assess the direct involvement of GR in cardiomyocytes we used SMGRKO mice, with *SM22-Cre*-driven deletion of GR in cardiomyocytes and VSMC. The effect on heart function *in utero* was investigated at E15.5 and at E17.5, during and 2 days following cessation of treatment, respectively. Parameters of glucocorticoid action were investigated *ex vivo*, including GR (*Nr3c1*) and GR target genes (*Fkbp5*, *Kcnj12*) as well as markers of cardiac maturation – genes encoding calcium-handling proteins that are indirectly regulated by GR in heart (Rog-Zielinska *et al.* 2015).

## Materials and methods

### Animals

All animal experiments were approved by the University of Edinburgh Animal Welfare and Ethical Review Body and were carried out in strict accordance with accepted standards of humane animal care under the auspices of the Animal (Scientific Procedures) Act UK 1986. SMGRKO mice (on a C57Bl/6 genetic background) have been previously described (Rog-Zielinska *et al.* 2013). They carry a deletion of exon 3 of the *Nr3c1* gene (encoding GR) in cardiomyocytes and vascular smooth muscle, the result of *Sm22-Cre* mediated recombination of a ‘floxed’ GR allele (*GR^fl/fl^*). Experimental SMGRKO fetuses and control littermates were generated by mating male SMGRKO (*GR^fl/fl ^Sm22-Cre^+^*) mice with control females (*GR^fl/fl ^Sm22-Cre^−^*). The morning of the day the vaginal plug was found was designated E0.5. Singly housed pregnant females received either dexamethasone (100 μg/kg/day, dose calculated based on daily weight and water intake measurements per mouse) (Sigma-Aldrich) or vehicle (EtOH, approx 0.023% depending on body weight and water consumption) in their drinking water from E12.5 to E15.5. Cre genotyping was carried out on tissue biopsies using primers: 5′-GATCGCTGCCAGGATATACG-3′ and 5′-AGGCCAGGTATCTCTGACCA-3′. Fetuses were weighed and then killed by decapitation at either E15.5 or E17.5. Fetal heads were rapidly frozen on dry ice for *in situ* mRNA hybridisation. For RNA extraction, fetal hearts were dissected, weighed and rapidly frozen on dry ice. For liquid chromatography-mass spectrometry (LC-MS/MS), fetal and maternal livers were dissected, weighed and rapidly frozen on dry ice. Tissues were stored at −80°C prior to extraction and analysis.

### High-resolution ultrasound analysis

Fetal cardiac function was assessed at E15.5 or E17.5 using a Visualsonics Vevo 770 High-Resolution Ultrasound Scanner. Briefly, pregnant mice were anaesthetised with 2% isoflurane gas, laid supine with all limbs taped to electrocardiogram electrodes to monitor heart rate (~450 bpm) and body temperature was maintained at 37°C. Fetuses were counted for identification purposes and all were scanned. Abdominal hair was removed using a commercially available depilating cream, then pre-warmed ultrasound gel (Aquasonic 100; Parker Laboratories, Orange, NJ, USA) was applied. A 30 MHz RMV 707B (real-time microvisualisation) transducer was used, with a focal length of 12.7 mm and a depth of field view of 20 mm. Pulse-wave Doppler was used to measure blood flow across the mitral valve using the apical four-chamber view. Vevo 770 image analysis software was used to measure and analyse B-mode images and Doppler waveform traces. Following imaging, the dam was killed by cervical dislocation. Scanned fetuses were excised following identification by validation of position from the scanned images, then weighed, decapitated and tissues were collected.

### RNA analysis

RNA was extracted from fetal hearts by homogenisation in TRIzol (Thermo Fisher Scientific) followed by purification using a Purelink RNA mini kit (Thermo Fisher Scientific). cDNA was synthesised using a QuantiTect Reverse Transcription Kit (Qiagen) and then subject to quantitative (q)PCR (in triplicate) using the Roche Lightcycler 480 system with gene-specific primer sets and Universal Probe Library (Roche) (Supplementary Table 1, see section on supplementary data given at the end of this article). A standard curve was prepared from pooled cDNA samples. Relative quantification was provided by LightCycler software using the maximum second derivative method. mRNA levels are expressed relative to a combination of the mean of 2 or more of *18s*, *Actb*, *Hprt* and *Tbp* mRNA levels, used as internal standards (as detailed in the figure legends).

### *In situ* mRNA hybridisation

Coronal sections (10 µm) of fetal heads were cut using a cryostat, collected onto Polysine slides and stored at −75°C until processing. Corticotropin-releasing hormone (*Crh*) mRNA was measured using a ^35^S-UTP radiolabelled cRNA probe used extensively by ourselves and others (Welberg *et al.* 2000, Harris *et al.* 2001, Brunton *et al.* 2015, Grundwald & Brunton 2015) under conditions optimised in our laboratory. Briefly, ^35^S-cRNA was synthesised from a linearised pBluescript vector expressing a 518 bp *Crh* cDNA fragment (a gift from Dr Robert Thompson, University of Michigan, Ann Arbor, MI, USA). The plasmid was linearised with *HindIII* or *XbaI* and transcribed from the T7 or T3 promoters, respectively to synthesise the sense and antisense probes. ISH was performed as previously described (Brunton *et al.* 2009), except that following the RNase A step, slides were washed 3 × 50 min in 0.1 × SSC at 60°C. Following the post-hybridisation washes, sections were dehydrated in an ascending series of ethanol containing 300 mM ammonium acetate, air-dried and then exposed to autoradiographic film (Amersham Hyperfilm MP; GE Healthcare) at room temperature. Films were developed (Ilford Phenisol) and fixed (Ilford Hypam fixer). To optimise exposure times for autoradiographic film, a trial set of slides were exposed to film and developed at 2-day intervals until the exposure time which produced the best signal-to-noise ratio was identified and this exposure time (6 days) was used for the experimental tissue. Slides were subsequently dipped in autoradiographic emulsion and exposed at 4°C for 5 weeks then developed, counterstained with haematoxylin and eosin and viewed under a light microscope to confirm neuroanatomical localisation of the PVN. CRH mRNA expression in the paraventricular nucleus (PVN) of the hypothalamus was examined bilaterally in 3–6 sections/mouse with measurements taken from the mid-section on which CRH was apparent. Semiquantitative densitometric analysis of autoradiographic films using ImageJ software was used to calculate mean grey density. Sections hybridised with sense probes served as negative controls and showed no signal above background.

### Western blot

Protein was extracted from fetal heart tissue by homogenising in RIPA lysis and extraction buffer, followed by protein quantification using a Pierce BCA assay (both Thermo Fisher Scientific). Samples (20 μg protein) were added to NuPAGE Sample Reducing Buffer and NuPAGE LDS Sample Buffer (both Thermo Fisher Scientific) and denatured at 70°C for 10 min prior to electrophoresis on a NuPAGE Novex 4–12% Bis Tris gel in NuPAGE MES SDS running buffer (Thermo Fisher Scientific). Following protein transfer to a nitrocellulose membrane, non-specific binding was blocked by incubation in 5% blotting-grade blocker (BioRad) in TBS-T for 1 h. Membranes were incubated with primary antibodies: GR (1:400; G-5: sc-393232, Santa Cruz) and β-tubulin (1:1000; MAB3408, Merck MilliPore) overnight at 4°C. Membranes were then washed and incubated with secondary antibodies IRDye 800CW Goat anti-mouse IgG (1:10000; 926-32210, Licor Biosciences) and IRDye 680RD Goat anti-Mouse IgG (1:10,000; 925-68070, Licor Biosciences), followed by quantification of GR protein levels relative to β-tubulin using an Odyssey infrared imaging system (Licor Biosciences).

### Liquid chromatography-mass spectrometry

Livers were pooled from all fetuses from a litter to accumulate a weight of approximately 100 mg tissue (1 sample per litter). Tissues were homogenised in 3 mL methanol:water (7:2, v/v) and enriched with 5 ng internal standard solution (Epi-corticosterone d4-dexamethasone, d4-cortisol) added to each sample. Homogenates were shaken and then centrifuged at 3200 ***g*** for 45 min at 4°C before collecting the supernatant and reducing to dryness using oxygen-free nitrogen at 60°C. For simultaneous quantification of dexamethasone, 6-hydroxydexamethasone, 11-dehydrodexamethasone, corticosterone and 11-dehydrocorticosterone, calibration standards (1 mg/mL) of each steroid were diluted to 10 µg/mL stock in methanol to generate a calibration curve (range, 0.0001–50 ng, geometric progressions of 25) enriched with 5 ng internal standard solution (Epi-corticosterone d4-dexamethasone, d4-cortisol) and reduced to dryness under oxygen-free nitrogen. Samples and calibration standards were resuspended in 2 mL 20% methanol and transferred to Classic Sep-pak columns (2 mg, Waters, Borehamwood, UK) that had been primed with methanol (2 mL) and water (2 mL) before sample loading. Sep Paks were washed with 2 mL water and steroids were eluted with 2 mL 100% methanol and collected into a glass vial, before reducing to dryness under oxygen-free nitrogen at 60°C. Samples were resuspended in 100 µL 70:30 water/acetonitrile for LC-MS/MS analysis.

Samples were injected (20 µL) onto a C18-AR column (ACE Excel 150 × 2.1 mm; 2 µm, ACT Technologies, Aberdeen, UK) at 40°C using a flow rate of 0.5 mL/min, mobile phase A – water 0.1% formic acid, B – acetonitrile 0.1% formic acid from 20 to 90% B over 10 min, protected by a Kinetex KrudKatcher (Phenomenex, Macclesfield, UK), followed by analysis on a QTrap 5500 LC-MS/MS System (Sciex) or QTrap 6500 Triple Quadrupole, Mass Spectometer (Sciex) system.

The peak areas of each steroid and each internal standard were integrated using MultiQuant version 3.0.8 software (Version number 3.0.8664.0 2015 AB SCIEX with Scheduled MRM Algorithm support) (Sciex 2015). Linear regression analysis of calibration standards, calculated using peak area ratios of steroid of interest to internal standard, was used to determine the concentration of the steroid of interest in the samples. *R* ^2^ > 0.99 was considered acceptable and within each batch of samples the accuracy at the upper and lower limits were only accepted if accuracy <20%. The amount of steroid was calculated using linear regression analysis of the peak area ratio and was expressed as ng of analyte per g liver.

### Statistical analysis

All numerical data are presented as means ± s.e.m., with *P* < 0.05 deemed significant. GraphPad Prism 6 software was used for statistical analysis, with unpaired Student’s *t*-test and two-way ANOVA with *post hoc* Tukey or Bonferroni tests, as appropriate. All data were subject to Shapiro–Wilk normality testing prior to further analysis.

## Results

### Dexamethasone treatment reduced fetal number at E17.5, irrespective of genotype with no effect on heart weight. Body weight was increased in SMGRKO mice at E17.5

**Figure 1:**
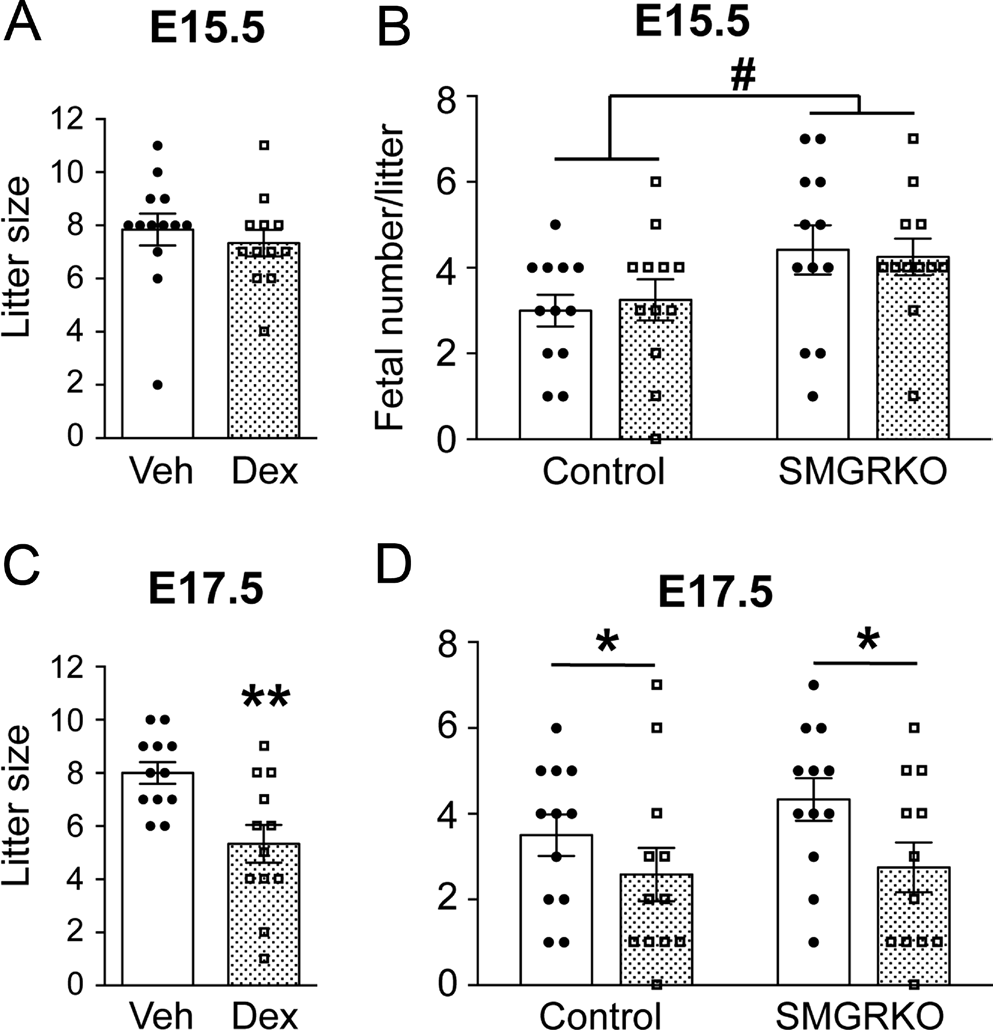
Dexamethasone treatment reduces fetal number and litter size at E17.5 without affecting either at E15.5. Dexamethasone (Dex, stippled bars: 100 µg/kg/day) or vehicle (Veh, white bars) was administered to pregnant dams in their drinking water from E12.5 to E15.5. Individual data points represent mean values per litter. Data are means ± s.e.m. and were analysed by Student’s *t-*test (A and C) or two-way ANOVA with *post hoc* Tukey tests (B and D). Significant effect of treatment, *P < 0.05, **P < 0.01; significant effect of genotype, ^#^*P* < 0.05, *n* = 12–13 litters per group.

Dexamethasone treatment from E12.5 to E15.5 had no effect on litter size at E15.5 (Fig. 1A). We noted that, irrespective of treatment, E15.5 litters contained more SMGRKO than control fetuses (Fig. 1B). There was no effect of dexamethasone on fetal weight at E15.5, nor did this differ between genotypes (Table 1), and there was no association between fetal weight and litter size (Supplementary Fig. 1A). Heart weight at E15.5 was unaffected by dexamethasone treatment and was similar between control and SMGRKO mice (Table 1). In contrast, 2 days after cessation of dexamethasone treatment, at E17.5, litter size was reduced (Fig. 1C). Both genotypes were affected (Fig. 1D), suggesting the decrease in litter size is not due to a direct effect of dexamethasone on the fetal heart via GR. Fetal weight did not show any relationship to litter size at E17.5 (Supplementary Fig. 1B), nor did we see any direct evidence for an increase in fetal deaths at E17.5 (2 dead/resorbed fetuses across all 24 litters at E17.5, vs 4 across 24 litters at E15.5). However, at E17.5, dexamethasone-treated SMGRKO fetuses weighed more than littermate controls and were also heavier than vehicle-treated SMGRKO fetuses (Table 1). This is suggestive of oedema in SMGRKO fetuses, as previously identified (Rog-Zielinska *et al.* 2013), 2 days following cessation of dexamethasone treatment; however, this requires experimental confirmation. There were no differences in fetal heart weights between genotypes or treatment groups at E17.5 (Table 1).

**Table 1:** Dexamethasone treatment increased body weight of SMGRKO fetuses at E17.5 but had no effect on fetal body weight or heart weight at E15.5.

	Vehicle		Dexamethasone	
	Control	SMGRKO	Control	SMGRKO
E15.5				
Body weight (mg)	420.7 ± 20.1	435.6 ± 18.0	459.0 ± 14.6	428.6 ± 14.9
Heart weight (mg)	12.4 ± 1.0	12.8 ± 0.7	13.7 ± 0.7	14.0 ± 0.9
Heart weight/body weight	0.034 ± 0.003	0.035 ± 0.002	0.036 ± 0.003	0.035 ± 0.003
E17.5				
Body weight (mg)	873.9 ± 14.3	850.8 ± 13.4	855.2 ± 22.0	954.5 ± 36.4**^,†^
Heart weight (mg)	13.1 ± 0.7	13.2 ± 0.7	13.8 ± 0.5	14.3 ± 0.12
Heart weight/body weight	0.015 ± 0.001	0.016 ± 0.001	0.017 ± 0.001	0.018 ± 0.002

Dexamethasone (100 µg/kg/day) or vehicle was administered to pregnant dams in drinking water, from E12.5 to E15.5. Fetuses were collected at E15.5 or E17.5. Fetal body and heart weights are in mg with heart weight also expressed relative to body weight. Data are means ± s.e.m. and were analysed using two-way ANOVA with *post hoc* Tukey’s test. Body weight: *n* = 29–52 fetuses from 5 to 12 litters/treatment. Heart weight: *n* = 11–28 fetuses from 5 to 6 litters/treatment.

**P < 0.01 vs untreated SMGRKO; ^†^*P* < 0.05 vs dexamethasone-treated controls.

### Dexamethasone treatment transiently altered cardiac diastolic function at E15.5 in a GR-dependent manner, with no effect on systolic function

**Figure 2:**
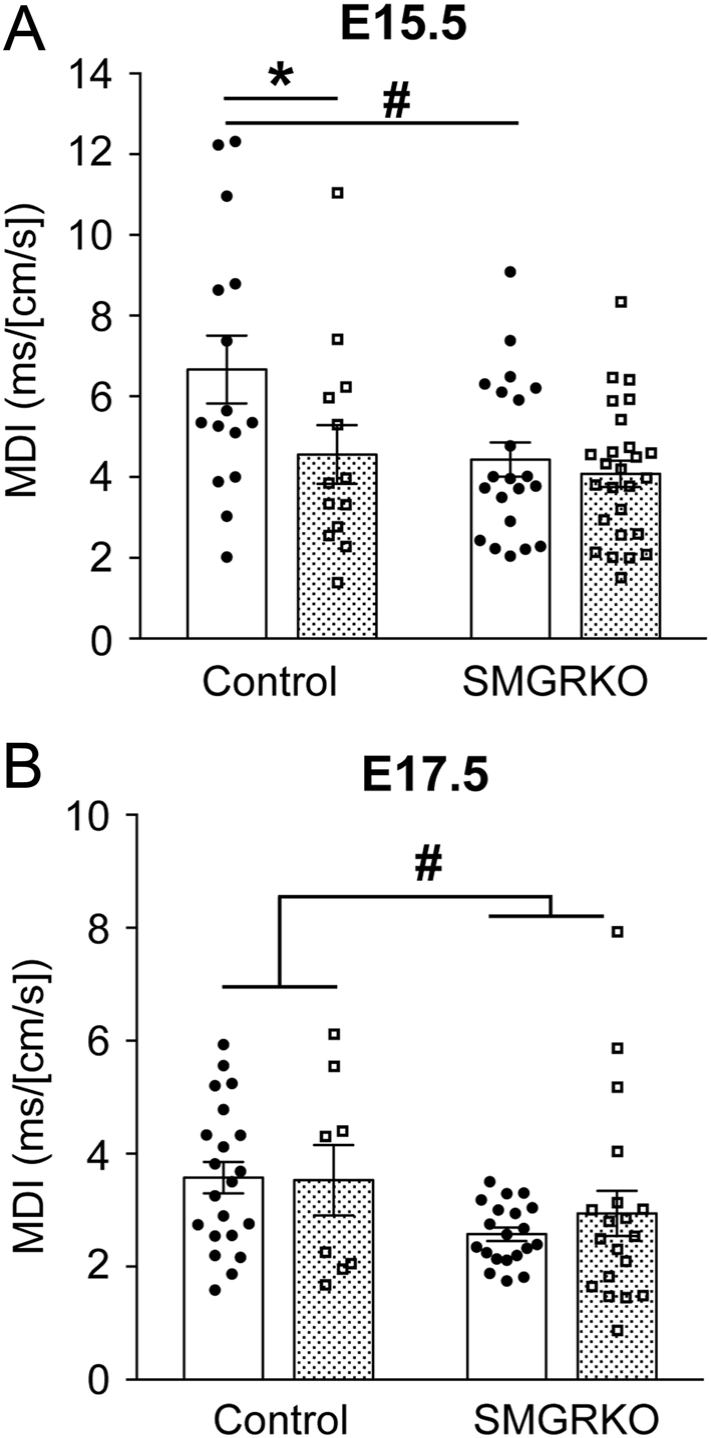
Dexamethasone transiently reduces the mitral deceleration index, dependent on GR. Dexamethasone (100 µg/kg/day; stippled bars) or vehicle (Veh, white bars) was administered to pregnant dams from E12.5 to E15.5. Mitral deceleration index (MDI) was measured by *in vivo* ultrasound imaging at (A) E15.5 and (B) E17.5. Data are means ± s.e.m. and were analysed by two-way ANOVA with *post hoc* Tukey tests: significant effect of treatment, *P < 0.05; significant effect of genotype, ^#^*P* < 0.05, *n* = 8–28 fetuses from 5 to 6 litters per group.

Fetal adrenal glucocorticoid synthesis in mice initiates at E14.5 and peaks around E17.5 (Michelsohn & Anderson 1992). To investigate whether antenatal dexamethasone treatment prior to this can advance the maturation of fetal heart function, echocardiography was performed at E15.5 (end of treatment) or at E17.5 (2 days after cessation of treatment). Heart rate was not significantly affected by genotype or treatment at either time point and was in the normal range for these gestational ages (Supplementary Table 2) (Corrigan *et al.* 2010). There was no difference in the Doppler-derived myocardial performance index (MPI – a measure of combined systolic and diastolic function) between genotypes at either E15.5 or E17.5, nor was it altered by dexamethasone at either time point. There were also no effects of dexamethasone on any of the components of MPI (IVCT, IVRT, ET), although the IVRT showed the expected maturational decrease between E15.5 and E17.5, irrespective of dexamethasone treatment (Supplementary Table 2). The Doppler-derived E/A wave ratio (the ratio of peak blood flow velocity in early ‘passive’ diastole to peak velocity in late ‘active’ diastole) was also unaffected by dexamethasone treatment, nor did it differ between genotypes (Supplementary Table 2). However, at E15.5, compared to control mice, SMGRKO mice showed a lower mitral deceleration index (MDI), calculated by normalising the early filling deceleration time (DT) for peak E wave velocity. This suggests altered diastolic heart function. Moreover, whilst the MDI of SMGRKO fetuses was unaffected following dexamethasone treatment, dexamethasone decreased the MDI in control mice to a level similar to that of SMGRKO mice (Fig. 2A), suggesting MDI is responsive to GR activation in cardiomyocytes/VSMC. By E17.5, the MDI of dexamethasone-treated control fetuses had recovered to the same level as in vehicle-treated controls, although the reduction in the MDI in SMGRKO mice compared to controls persisted (Fig. 2B).

### Calcium-handling genes, markers of fetal heart maturation, are downregulated by dexamethasone treatment

In their report of the application of high-frequency ultrasound to assess the maturation of the mouse fetal heart, Corrigan *et al*. failed to detect a change in MPI between E16.5 and E18.5 (Corrigan *et al.* 2010, Rog-Zielinska *et al.* 2013), suggesting that MPI alone might not be a reliable marker of heart maturation. We therefore examined the expression of genes involved in calcium handling that normally increase during fetal heart maturation and which are indirectly induced by GR in fetal cardiomyocytes (Rog-Zielinska *et al.* 2015). At E15.5, 2-way ANOVA showed a significant effect of dexamethasone in reducing cardiac expression of *Atp2a2*, *Cacna1c* and *Slc8a1* (encoding SERCA2a, Ca_v_1.2 and NCX1, respectively); *Ryr2* was unaffected (Supplementary Fig. 2A). By E17.5, expression had recovered in dexamethasone-exposed fetuses, with *Cacna1c* even increased in dexamethasone-exposed control fetuses (Supplementary Fig. 2B). Overall, these findings are not consistent with an advancement in fetal heart maturation, and moreover, suggest that dexamethasone may transiently impair fetal heart function by reducing capacity for calcium handling. This occurred in both genotypes and is therefore independent of GR expression in cardiomyocytes/VSMC.

### Glucocorticoid signalling in fetal heart may be downregulated by dexamethasone treatment

**Figure 3:**
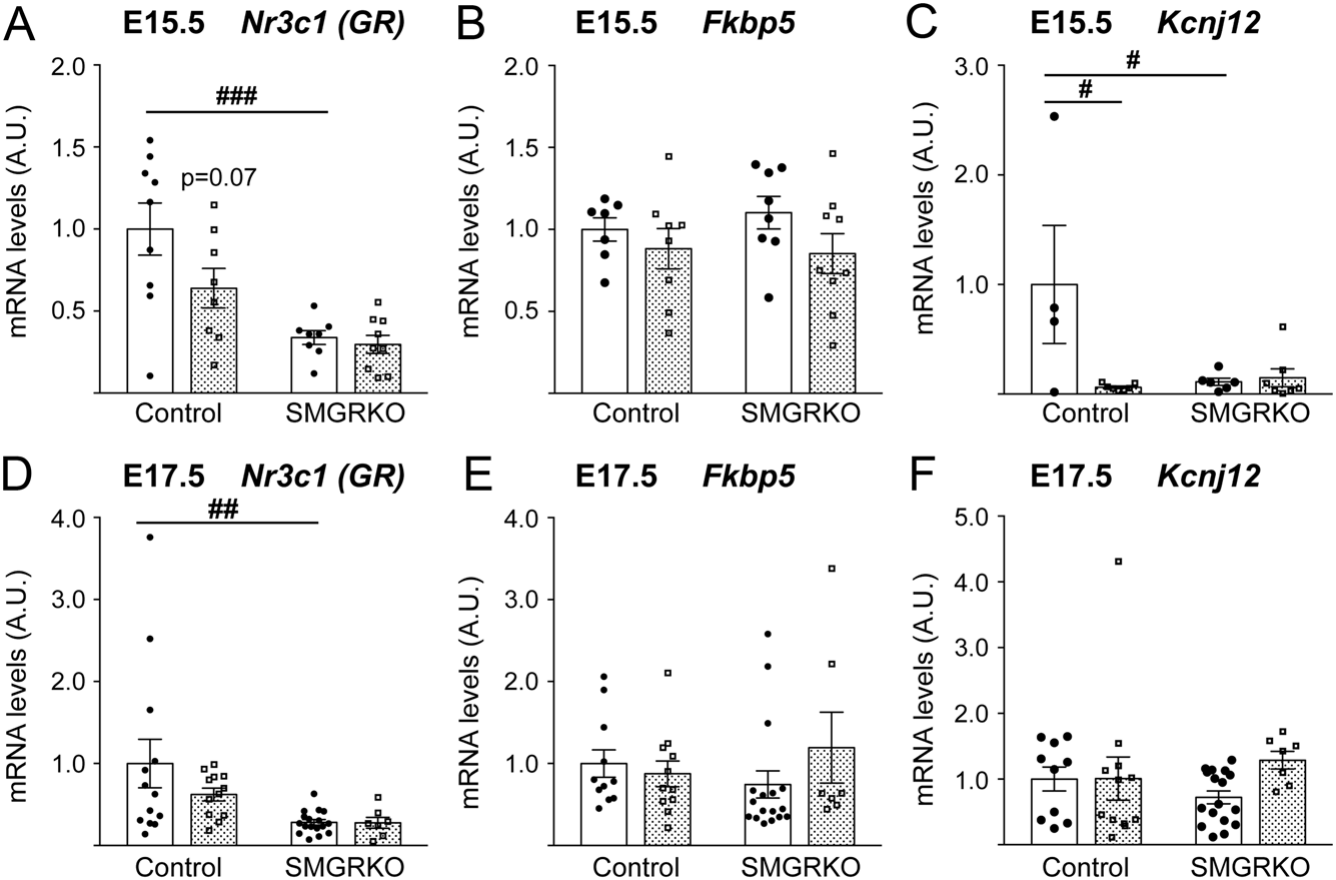
Glucocorticoid signalling in hearts of control fetuses is downregulated by dexamethasone at E15.5. Dexamethasone (100 µg/kg/day, stippled bars) or vehicle (Veh, white bars) was administered to pregnant dams in their drinking water from E12.5 to E15.5. qRT-PCR measurements at E15.5 of cardiac levels of (A) *Nr3c1* mRNA, (B) *Fkpb5* mRNA, (C) *Kcnj12* mRNA, all relative to the mean of *Actb*, *Hprt* and *Tbp* mRNA levels, and at E17.5 of (D) *Nr3c1* mRNA, (E) *Fkbp5* mRNA and (F) *Kcnj12* mRNA, all relative to the mean of *18s*, *Hprt* and *Tbp* mRNA levels. Data, in arbitrary units (A.U.), are means ± s.e.m. and were analysed by two-way ANOVA with *post hoc* Tukey tests (^###^*P* < 0.001, ^##^*P* < 0.01, ^#^*P* < 0.05 vs vehicle-treated control mice), *n* = 7–17 fetuses from six litters per group.

The decrease in MDI following dexamethasone treatment in control fetuses at E15.5, to the same level as that in SMGRKO fetal hearts, suggests that dexamethasone may have downregulated GR signalling in control fetal hearts, causing MDI (a dynamic measure) to be equivalent to that in the GR-deficient SMGRKO hearts. Consistent with this idea, levels of *Nr3c1* mRNA (encoding GR) in control fetal hearts tended to be reduced (*P* = 0.07) following 3 days dexamethasone treatment (Fig. 3A). There was also a non-significant trend for downregulation of GR protein levels in hearts of control fetuses at E15.5 following dexamethasone treatment (Supplementary Fig. 3). As expected, *Nr3c1* mRNA levels were lower in SMGRKO fetal hearts at E15.5 compared to control littermates and were unaffected by dexamethasone (Fig. 3A). By E17.5, 2 days after cessation of dexamethasone treatment, cardiac *Nr3c1* mRNA levels had largely recovered in control fetuses (Fig. 3D). Levels of *Fkbp5* mRNA, a glucocorticoid target gene, did not show the expected increase with dexamethasone treatment in control fetal hearts at E15.5, nor were they different in SMGRKO hearts (Fig. 3B). At E17.5, *Fkbp5* mRNA levels remained unaffected by prior dexamethasone treatment (Fig. 3E). Similarly, *Kcnj12*, a direct target of GR in fetal cardiomyocytes (Rog-Zielinska *et al.* 2015), was downregulated by dexamethasone in control fetuses at E15.5 (Fig. 3C), with recovery of normal levels by E17.5 (Fig. 3F). These data are consistent with an acute downregulation of glucocorticoid signalling following 3 days of dexamethasone treatment.

### Fetal glucocorticoid levels are altered by dexamethasone treatment

**Figure 4:**
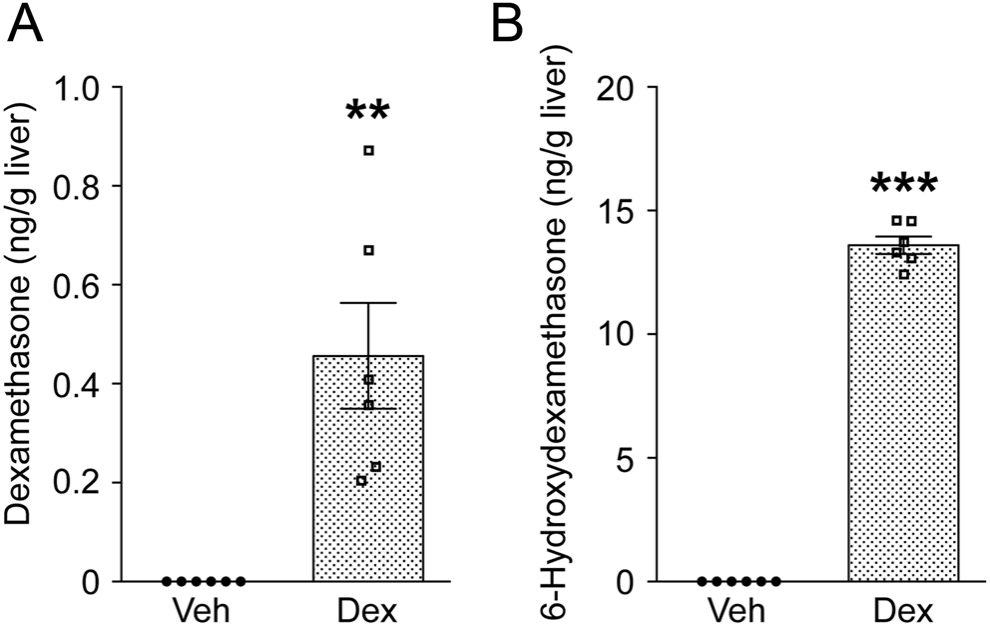
Dexamethasone and its metabolite 6-hydroxydexamethasone are present in livers of dexamethasone-treated fetuses at E15.5. Dexamethasone (Dex, stippled bars: 100 µg/kg/day) or vehicle (Veh, white bars) was administered to pregnant dams from E12.5 to E15.5, with no further treatment to E17.5. Dexamethasone (A) and 6-hydroxydexamethasone (B) were measured in fetal livers pooled from each litter (approximately 100 mg tissue), by mass spectrometry. The detectable range for dexamethasone was 0.01–5 ng and for 6-hydroxydexamethasone 0.5–25 ng. Values are expressed as ng steroid/g fetal liver. Each data point represents a single pool of fetal livers from one litter. Data are means ± s.e.m. and were analysed by unpaired Student’s *t-*test (**P < 0.01, ***P < 0.001), *n* = 4–6.

To establish that dexamethasone reached the fetal circulation and to determine its effects on endogenous glucocorticoid concentrations, the level of dexamethasone and its major metabolites 6-hydroxydexamethasone and 11-dehydrodexamethasone were measured. We have previously measured corticosterone levels in the mid and late gestation fetal heart (Rog-Zielinska *et al.* 2013), in which the rise in glucocorticoid levels reflected the late gestation rise in plasma and tissue glucocorticoid levels. Because we had limited amounts of heart tissue, we reasoned that the concentration of steroids in liver, as an important target of glucocorticoid action as well as steroid and drug metabolism, would be indicative of overall exposure. Fetal livers were pooled from each litter to obtain sufficient sample for mass spectrometry measurement of steroid concentration. The endogenous glucocorticoids, corticosterone and 11-dehydrocorticosterone (11-DHC) were simultaneously measured. At E15.5, dexamethasone was detected in the livers of treated fetuses, though at lower levels than 6-hydroxydexamethasone (Fig. 4A and B). 11-Dehydrodexamethasone was undetectable (over a dynamic range of assay, 0–10 ng). By E17.5, neither dexamethasone nor its metabolites were detectable in fetal livers, nor were they present in vehicle-treated fetuses at either time point (data not shown). Similarly, dexamethasone and 6-hydroxydexamethasone were detected in the livers of dexamethasone-treated pregnant dams at E15.5 (Supplementary Fig. 4), but not at E17.5, whilst 11-dehydrodexamethasone was undetectable (data not shown).

**Figure 5:**
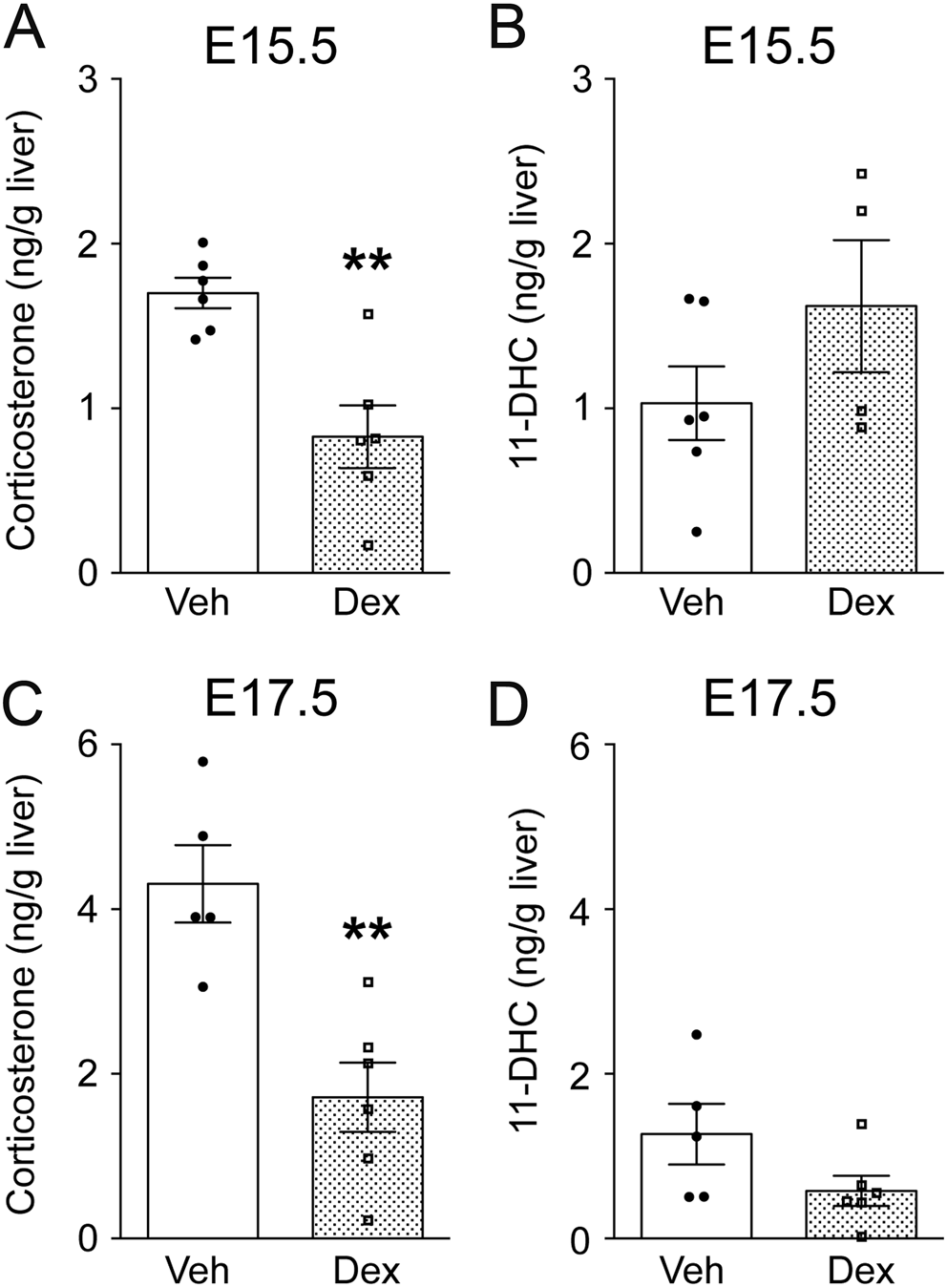
Suppression of corticosterone levels in fetal liver following dexamethasone treatment. Dexamethasone (Dex, stippled bars: 100 µg/kg/day) or vehicle (Veh, white bars) was administered to pregnant dams from E12.5 to E15.5, with no further treatment to E17.5. Steroids were measured in fetal livers pooled from each litter (approximately 100 mg tissue), by mass spectrometry. The upper and lower limits of detection of both corticosterone and 11-dehydrocorticosterone (11-DHC) was 0.05–10 ng. Levels of (A) corticosterone at E15.5, (B) 11-DHC at E15.5, (C) corticosterone at E17.5 and (D) 11-DHC at E17.5. Values are expressed as ng steroid/g fetal liver with each data point representing a single pool of fetal livers from one litter. Data are means ± s.e.m. and were analysed by unpaired Student’s *t-*test; **P < 0.01, *n* = 4–6.

Consistent with the low glucocorticoid environment of the fetus, corticosterone levels were low in pools of fetal livers at E15.5 and were reduced by dexamethasone treatment (Fig. 5A). Corticosterone levels remained suppressed in dexamethasone-exposed fetuses at E17.5, 2 days after cessation of treatment (Fig. 5C). In contrast, 11-dehydrocorticosterone levels were not significantly affected by dexamethasone treatment at either time point (Fig. 5B and D). Corticosterone levels in the liver of the dam were much higher than those in the fetus, and although hepatic corticosterone levels in the dam tended to be reduced following dexamethasone treatment, this was not statistically significant (Supplementary Fig. 5). Fetal hepatic 11-dehydrocorticosterone concentrations were an order of magnitude lower than corticosterone in the dams and were reduced by dexamethasone treatment at E15.5. However, levels had recovered by E17.5. These data could reflect hypothalamic–pituitary–adrenal (HPA) axis suppression, reducing levels of corticosterone and/or 11-dehydrocorticosterone (plasma levels of 11-dehydrocorticosterone increase following HPA axis activation; Kotelevtsev *et al.* 1997, Harris *et al.* 2001, Verma *et al.* 2018). Alternatively, alterations in absolute or relative activities of maternal or fetal 11β-hydroxysteroid dehydrogenases, which interconvert active and intrinsically inert glucocorticoids (Chapman *et al.* 2013), could account for the effects of dexamethasone on endogenous glucocorticoid levels. To try to distinguish between these possibilities, we carried out *in situ* mRNA hybridisation to measure levels of *Crh* mRNA in the fetal paraventricular nucleus of the hypothalamus. However, whilst the expected *Crh* downregulation was seen in dexamethasone-treated SMGRKO fetuses at E15.5, there was no significant effect in control fetuses at this age, nor were there any significant treatment differences between fetuses at E17.5 (Supplementary Fig. 6). Thus, the reduced corticosterone levels in dexamethasone-treated fetuses may result from altered placental and/or fetal glucocorticoid metabolism and/or transport. Alternatively, they may be a consequence of changes in pituitary and/or adrenal sensitivities, which were not assessed here.

## Discussion

Contrary to our hypothesis, administration of dexamethasone from E12.5 to E15.5 does not advance fetal heart maturation in mice. MPI, a marker of fetal heart maturity (Corrigan *et al.* 2010, Rog-Zielinska *et al.* 2013), was not significantly affected by antenatal dexamethasone, nor were calcium-handling genes induced. Heart function was altered though. Dexamethasone transiently decreased the MDI in control fetuses, with recovery by 2 days after cessation of treatment. Other parameters of diastolic function (DT, E/A Doppler velocity ratio, IVRT) were not significantly affected by dexamethasone. There was no effect of dexamethasone on the MDI in SMGRKO fetuses. In an adult population with a high prevalence of hypertension and diabetes but free of prevalent cardiovascular disease, MDI predicted cardiovascular events (Mishra *et al.* 2007). In another human study, MDI was significantly associated with age and aortic root diameter (Cuspidi *et al.* 2011). However, the impact of an alteration in MDI during development is unknown, both in the fetus and longer-term in adulthood. Thus, whether the reduction in the MDI in dexamethasone-treated control fetuses is detrimental, beneficial or simply reflects different loading conditions, is currently unclear. Diastolic function is strongly dependent on haemodynamic status and alterations in either preload or afterload affect mitral flow velocity curves (Nishimura *et al.* 1989*a*,*b*). The U-shaped relationship between DT and cardiovascular outcomes is strengthened by normalising for the E wave velocity, thereby correcting for loading conditions in calculating the MDI (Mishra *et al.* 2007, Chinali *et al.* 2009). Here, DT itself was unaffected by dexamethasone. This suggests that the effect of dexamethasone on the MDI in control fetuses is likely due to alterations in loading, possibly through the well-known increase in fetal blood pressure following antenatal corticosteroid administration (Derks *et al.* 1997, Bennet *et al.* 1999). In the surviving dexamethasone-treated control fetuses, MDI was normalised by E17.5. Although diastolic dysfunction in human fetuses with cardiomyopathy is associated with an eight-fold increased risk of fetal mortality compared to systolic dysfunction (Pedra *et al.* 2002), it is unlikely that any diastolic dysfunction reflected in the lower MDI at E15.5 contributes to the reduced fetal survival at E17.5. Dexamethasone treatment has a similar effect in reducing the number of SMGRKO and control fetuses at E17.5; yet, the MDI in SMGRKO mice is unaffected by dexamethasone. Nevertheless, it is possible that systemic factors, particularly haemodynamic, differentially affect diastolic function in SMGRKO and control fetuses. The increase in wet weight at E17.5 in SMGRKO fetuses with prior dexamethasone treatment is consistent with our previous finding of mild oedema in untreated SMGRKO fetuses at E17.5 (Rog-Zielinska *et al.* 2013) and suggests genotype-specific cardiac and/or haemodynamic effects of dexamethasone. Whether dexamethasone causes or exacerbates oedema in SMGRKO fetuses requires experimental confirmation. SMGRKO mice have normal blood pressure as adults (Richardson *et al.* 2017). However, in an independent line of mice with GR deletion in VSMC (as in our SMGRKO mice), dexamethasone-induced hypertension is attenuated in adulthood (Goodwin *et al.* 2008). Whether GR action in VSMC contributes to the fetal heart phenotype observed here remains to be established.

The lack of difference in MPI between genotypes and with dexamethasone treatment, was surprising, as was the lack of genotype difference in calcium-handling gene expression, at least at E17.5. We have previously described an increase in MPI at E17.5 in SMGRKO fetuses compared with controls, and SMGRKO fetuses have lower cardiac expression of *Atp2a2* and *Ryr2* than littermate controls at E17.5 (Rog-Zielinska *et al.* 2013). The reason we failed to see those differences here is unclear; a reduction in cardiac GR expression in SMGRKO mice was confirmed. However, the SMGRKO mice used here have undergone several generations of breeding since the original line was generated and characterised, and it is possible that compensatory genetic changes have occurred (e.g. due to selection of surviving Cre+ male mice from which to breed), accounting for these findings. It is also possible that the difference in experimental conditions (e.g. daily weighing of the mice or factors unknown to us that alter haemodynamic status) could account for the discrepancies between this and our previous study. Ongoing and future studies will investigate this.

Nevertheless, a genotype-specific impact of dexamethasone upon the fetus is evident in this study. Our steroid measurements confirm dexamethasone in fetal livers, albeit at a concentration ~10-fold lower than in the maternal liver. This is consistent with previous reports of a gradient of this magnitude between maternal and fetal serum in the late gestation rat (Varma 1986). Interestingly, levels of 6-hydroxydexamethasone, an active metabolite of dexamethasone (Ritchie *et al.* 1992), are at least as high in the fetal liver as in the maternal liver. This relatively high level of 6-hydroxydexamethasone in the fetal liver suggests metabolism of dexamethasone to 6-hydroxydexamethasone by the feto-placental unit. This is the first known example of evidence for 6-hydroxydexamethasone expression in the mouse fetal liver.

Because, at E15.5, dexamethasone had no effect on the MDI in SMGRKO fetuses, but decreased the MDI in control fetuses (to be the same as in SMGRKO), we hypothesised that GR was downregulated in cardiomyocytes and/or VSMC of control mice after the 3 days of dexamethasone treatment. Downregulation of GR in response to dexamethasone treatment is well known (Wilkinson *et al.* 2018) and lower GR expression in the heart of control animals may explain the lack of dexamethasone effect on *Fkbp5*, a well-known GR target gene as well as *Kcnj12*, a GR target in cardiomyocytes (Rog-Zielinska *et al.* 2015). Any reduction in glucocorticoid sensitivity due to reduced GR expression is transient, recovering 2 days after termination of dexamethasone administration. Genes involved in calcium handling are normally induced following GR activation in cardiomyocytes, albeit by indirect mechanisms. The transient decrease at E15.5 with recovery by E17.5 is also consistent with downregulation of GR. However, the decrease in GR that impacts calcium handling is likely not in cardiomyocytes/VSMC, as calcium handling was also affected by dexamethasone in SMGRKO mice. The locus of this regulation of cardiac gene expression by dexamethasone is currently unclear. Dexamethasone treatment also perturbs endogenous fetal glucocorticoid levels, and the effect on the HPA axis appears complex. Prolonged suppression of the fetal HPA axis is suggested by the decrease in fetal corticosterone concentration without a significant change in 11-dehydrocorticosterone following dexamethasone treatment to E15.5 and persisting 2 days after cessation of treatment at E17.5. Whether or not this suppression of the fetal HPA axis persists beyond E17.5 was not investigated here, though antenatal glucocorticoid exposure is known to exert long-term programming effects upon HPA axis activity (Rog-Zielinska *et al.* 2014, Agnew *et al.* 2018). Contrary to expectations, *Crh* mRNA was not downregulated by dexamethasone in control animals, though it was in SMGRKO fetuses at E15.5, with recovery observed by E17.5. Because, for methodological reasons, fetal livers from both genotypes were pooled for steroid measurements, we could not determine whether hepatic corticosterone levels in the SMGRKO and control livers were similarly affected by dexamethasone treatment. Thus, it remains possible that there is a differential effect of dexamethasone upon fetal corticosterone levels in SMGRKO and control fetuses, as well as upon the HPA axis. Whilst speculative, this naturally raises the question of how HPA axis suppression could arise in SMGRKO but not control fetuses. It has been suggested that resistance to negative feedback by glucocorticoids is essential to allow the late gestation increase in glucocorticoid levels (Matthews & Challis 1996). CRH is not suppressed by antenatal betamethasone treatment in the late gestation sheep fetus, despite reduced cortisol levels (Li *et al.* 2013). In contrast, in the late gestation guinea pig fetus, CRH expression is reduced by repeated injection of synthetic glucocorticoids, demonstrating sensitivity to negative feedback (McCabe *et al.* 2001). Interestingly, in the late gestation fetal sheep hypothalamus, basal CRH synthesis is resistant to hypercortisolaemia, whereas increased CRH synthesis in response to hypoxia is highly sensitive to glucocorticoid negative feedback (Matthews & Challis 1995*a*,*b*). Whether such a mechanism operates in mice requires investigation. CRH synthesis is sensitive to haemodynamic variation in the fetus (Ng 2000), and conceivably, may be differentially regulated in SMGRKO and control fetuses, if haemodynamic forces differ between the genotypes in response to dexamethasone. It is also possible that sex differences may account for some of the differential effects. For example, the HPA axis of male guinea pig fetuses is more sensitive to negative feedback by maternally administered glucocorticoids at the pituitary level than that of the female fetuses (Owen *et al.* 2005). Males and females were not distinguished here.

Dexamethasone reduces survival of both SMGRKO and control fetuses at E17.5 (2 days after cessation of dexamethasone treatment). The cause of death is currently unknown, but plausibly could relate to reduced capacity for calcium handling, which was affected in both genotypes. It is possible that the dexamethasone-related deaths have biased our findings at E17.5, if deaths occurred in more severely impacted fetuses; indeed, a limitation of the current study is that data from all fetuses were included, irrespective of litter size. Thus, the analysis could possibly have been subject to maternal bias. Of note, in a large clinical study in low- and mid-income countries, antenatal dexamethasone treatment increased the incidence of neonatal death (Althabe *et al.* 2015). The risk was greatest in those babies born close to term and importantly, only 16% of the women who received antenatal corticosteroid actually went on to deliver their baby preterm. This raises concerns about possible harmful effects of non-optimally administered antenatal corticosteroids. Our study is consistent with these clinical data and suggests cessation of glucocorticoid treatment confers an increased vulnerability to sudden death. Whilst undoubtedly life saving when appropriately administered, many women administered antenatal corticosteroids in high-income countries do not deliver their babies within 1–7 days following treatment (reviewed in Kemp *et al.* 2018). This highlights a need for further research to fully understand the consequences of antenatal glucocorticoid administration to inform and refine antenatal corticosteroid therapy.

In sheep, late gestation maternal hypercortisolemia causes perinatal death (Keller-Wood *et al.* 2014), associated with cardiac arrhythmias and decreased fetal aortic pressure (Antolic *et al.* 2018). Thus, there appears an inverted U-shaped relationship between glucocorticoid levels and fetal survival with both glucocorticoid deficiency and glucocorticoid excess reducing perinatal survival. At least some of this effect is likely to be mediated by the complex effects of glucocorticoids on haemodynamic parameters and cardiac function described by us (Rog-Zielinska *et al.* 2013, Gray *et al.* 2017, Richardson *et al.* 2017), and by others (Reini *et al.* 2008, Feng *et al.* 2013, Oakley *et al.* 2013, Richards *et al.* 2014, Antolic *et al.* 2018). Unravelling these complex relationships, and the impact of genetic variation in glucocorticoid signalling upon fetal haemodynamic function, will be important for future therapy in preterm birth and in managing the consequences of extreme maternal stress. Effects on placental function were not investigated here. However, placental vascularisation and haemodynamic forces are critical regulators of cardiac maturation (Morrison *et al.* 2007, Thornburg *et al.* 2010, Burton *et al.* 2016). Moreover, recent data in *Hsd11b2^−^^/^^−^* mice suggest pharmacological rescue of impaired placental vasculature also rescues normal fetal cardiac maturation (Wyrwoll *et al.* 2016). Future research should address the impact of synthetic glucocorticoid administration upon placental vasculature and haemodynamics, in conjunction with effects on heart.

The long-term consequences of the dexamethasone treatment utilised here were not examined. However, in humans and in animals, there is an association between excessive glucocorticoid exposure *in utero* and an increased risk of cardiovascular disease in adult offspring (reviewed in Rog-Zielinska *et al.* 2014, Agnew *et al.* 2018). There are relatively few studies of the effects of antenatal glucocorticoid treatment upon adult cardiovascular disease risk in mice. A previous study in which mice were administered dexamethasone from E12.5 to E15 reported growth restriction and a trend for decreased heart weight at E14.5, with catch up by E17.5. As adults, the dexamethasone-exposed mice had normal hearts, but increased blood pressure and pulse pressure compared to controls (O’Sullivan *et al.* 2013). Mice are born relatively immature compared to humans. However, the scant evidence available suggests that early postnatal cardiomyocyte maturation, including capacity for proliferation and heart repair, is similar in mice and humans. In both humans and mice, there is a period in which cardiomyocytes continue to proliferate after birth (albeit possibly over a longer period in humans than the 1 week postnatally in mice) (Porrello *et al.* 2011, Mollova *et al.* 2013, Bergmann *et al.* 2015, Haubner *et al.* 2016, Yutzey 2017). Whether mid-to-late gestation cardiomyocyte maturation is similar in humans and mice is currently unknown. Our data, however, suggest that even transient effects of antenatal dexamethasone can interfere with the normal trajectory of cardiac maturation, possibly through altered haemodynamics, known to be affected by antenatal dexamethasone in humans (Mulder *et al.* 2009), thus potentially impacting on adult cardiovascular health. Thus, whilst antenatal glucocorticoids are undoubtedly of clinical benefit in infants born preterm, there may be detrimental effects on the heart and other organs, as seen here in an animal model. Future studies should establish the short- as well as long-term effects of antenatal corticosteroid therapy on human heart. The benefit *versus* risk balance must be carefully considered by clinicians before administering antenatal glucocorticoids. Further research is needed to optimise the timing and dosage to provide the most beneficial outcome overall.

## Supplementary Material

Supplementary Table 1. Primer sequences and the corresponding probes used for qRT-PCR analysis of mRNA.

Supplementary Table 2. Echocardiography parameters measured in E15.5 and E17.5 fetal mice.

Supplementary Figure 1. No association between mean fetal weight per litter and litter size, in vehicle and dexamethasone treated mice. Dexamethasone (Dex: 100µg/kg/day) or vehicle (Veh) was administered from E12.5 to E15.5. Individual data points represent mean fetal weight per litter (g). R2 values were as follows, (A) E15.5 vehicle: 0.127, (B) E15.5 dexamethasone: 0.041, (C) E17.5 vehicle: 0.01, (D) E17.5 dexamethasone: 0.009. n=10-12 litters.

Supplementary Figure 2. Levels of mRNA encoding calcium handing proteins are altered at E15.5 by dexamethasone treatment. Dexamethasone (100µg/kg/day, stippled bars) or vehicle (Veh, white bars) was administered to pregnant dams in their drinking water from E12.5 to E15.5. qRT-PCR measurements at (A) E15.5 and (B) E17.5 of cardiac levels of (left to right, respectively) Ryr2 mRNA (encoding RYR2), Atp2a2 mRNA (encoding SERCA2a), Slc8a1 mRNA (encoding NCX1) and Cacna1c mRNA (encoding Cav1.2). E15.5 measurements are relative to the mean of Actb, Hprt and Tbp mRNA levels and E17.5 are relative to the mean of 18s, Hprt and Tbp mRNA levels. Data, in arbitrary units (A.U.), are means ± SEM and were analysed by 2-way ANOVA with post-hoc Tukey tests. At E15.5, ANOVA showed a borderline significant genotype effect (p=0.054) for Ryr2, and significant effects of treatment for Atp2a2 (p<0.05), Slc8a1 (p<0.01) and Cacna1c (p<0.001). At E17.5, ANOVA showed a significant effect of treatment for Cacna1c (p<0.01). Post-hoc tests: ##p<0.01, #p<0.05, n=7-17 fetuses from 6 litters per group .

Supplementary Figure 3. GR protein levels in control fetal hearts at E15.5. Western blotting (representative image in upper panel) was used to measure protein levels in hearts of vehicle (V/Veh) and dexamethasone (D/Dex) treated mice. Graph shows quantification of GR protein levels relative to β-tubulin. Data are means ± SEM and were analysed by unpaired Student’s t-test, n= 4 (number of fetuses, from n=2-4 litters per group).

Supplementary Figure 4. Dexamethasone and its 6-hydroxydexamethasone metabolite are present in livers of dexamethasone-treated dams at E15.5. Dexamethasone (Dex: 100µg/kg/day) or vehicle (Veh) was administered from E12.5 to E15.5. Steroids were measured in maternal livers by mass spectrometry. The detectable range for (A) dexamethasone was 0.5-50ng, and for (B) 6-hydroxydexamethasone 0.1-5ng. Values are expressed as ng steroid/g liver. Data are means ± SEM and were analysed by unpaired Student’s t-test (*p<0.05, **p<0.01). Each data point represents a single dam, n=4-6.

Supplementary Figure 5. Endogenous glucocorticoid levels in dam liver at E15.5 and E17.5. Dexamethasone (Dex: 100µg/kg/day) or vehicle (Veh) was administered from E12.5 to E15.5. Steroid levels were measured in maternal livers by mass spectrometry: (A) corticosterone at E15.5, (B) 11-dehydrocorticosterone (11-DHC) at E15.5, (C) corticosterone at E17.5 and (D) 11-DHC at E17.5 . For E15.5 samples, the detectable range for corticosterone was 0.025-50ng and 11-DHC, 0.1-50ng. For E17.5 samples, the detectable range for corticosterone was 0.0025-10ng and 11-DHC, 0.0025-50ng. Values are expressed as ng steroid/g liver. Data are means ± SEM and were analysed by unpaired Student’s t-test,***p<0.001. Each data point represents a single dam, n=4-6.

Supplementary Figure 6. SMGRKO fetal HPA axis is downregulated at E15.5 following dex exposure, with no change at E17.5 or in the control ‘floxed’ group. Corticotropin releasing hormone (Crh) mRNA levels were measured bilaterally in the PVN of the hypothalamus by in situ hybridisation. (A) Representative images of autoradiographs of sections of fetal brain showing hybridisation of Crh mRNA in E15.5 (top panels) and E17.5 (bottom panels) control (left two panels) and SMGRKO (right 2 panels) fetal brains. Fetuses were vehicle or dexamethasone exposed, as indicated. (B, C) Densitometric quantification of Crh mRNA at (B) E15.5 and (C) E17.5. Levels of mRNA are expressed in arbitrary units (A.U.) with vehicle treated control set to 1. Data are means ± SEM and were analysed by 2-way ANOVA with post-hoc Tukey tests. (B) There was no significant effect of genotype or dexamethasone treatment individually, but there was a significant interaction (p=0.040). (C) Two-way ANOVA showed a significant effect (p=0.037) effect of genotype. n= 5-13 (number of fetuses, from n=2-5 litters per group).

## Declaration of interest

The authors declare that there is no conflict of interest that could be perceived as prejudicing the impartiality of the research reported.

## Funding

This work was supported by an MRC project grant (MR/P002811/1). E J A was supported by a scholarship from the British Heart Foundation (FS/13/52/30637). Additional support was provided by a British Heart Foundation Centre of Research Excellence Award (RE/08/001).

## Author contribution statement

Designed research: E J A, G A G, K E C. Execution: E J A, A G B, R V R, H M, A J W T, K S, G J, N Z M H, P B. Interpretation: E J A, A J W T, K S, G J, N Z M H, C M M, P B, G A G, K E C. Manuscript preparation: E J A, K E C.
