# Supplementary material for: Antenatal dexamethasone treatment transiently alters diastolic function in the mouse fetal heart
Source: J Endocrinol. 2019 Apr 23;241(3):279–92. doi: 10.1530/JOE-18-0666 (PMC6541236; doi:10.1530/JOE-18-0666)
Supplement: Supplementary Table 1. Primer sequences and the corresponding probes used for qRT-PCR analysis of mRNA. [file supplementary_table_1.pdf]

Supplementary Table 1. Primer sequences and the corresponding probes used for qRT-PCR analysis of mRNA.
Sequences of forward and reverse primers and the Universal Probe Library probe number used in qRT-PCR assays to measure levels of specific mRNAs.

**mRNA**	**Sequence 5'-3'**		**Probe**
	**Forward**	**Reverse**	
Actb	CTAAGGCCAACCGTGAAAAG	ACCAGAGGCATACAGGGACA	64
Atp2a2	TCGACCAGTCAATTCTTACAGG	CAGGGACAGGGTCAGTATGC	94
Cacna1c	CCTGCACAAGGGCTCTTTC	AGATGAGGGACACGCTAACC	62
Fkbp5	AAACGAAGGAGCAACGGTAA	TCAAATGTCCTTCCACCACA	97
Hprt	TCCTCCTCAGACCGCTTTT	CCTGGTTCATCATCGCTAATC	95
Kcnj12	GGCCTAGACCGTATCTTCCTG	TGGCCTCATCAATCTCGTG	9
Nr3c1	CAAAGATTGCAGGTATCCTATGAA	CTTGGCTCTTCAGACCTTCC	91
Ryr2	TTCAACACGCTCACGGAGTA	TGCCAGGCTCTGCTGATT	81
Slc8a1	GTCAGCCTTCAGAGCTGGTC	GACTTCCAACTGCTCCAACC	42
Tbp	GGGAGAATCATGGACCAGAA	GATGGGAATTCCAGGAGTCA	97
