# Supplementary material for: Antenatal dexamethasone treatment transiently alters diastolic function in the mouse fetal heart
Source: J Endocrinol. 2019 Apr 23;241(3):279–92. doi: 10.1530/JOE-18-0666 (PMC6541236; doi:10.1530/JOE-18-0666)
Supplement: Supplementary Table 2. Echocardiography parameters measured in E15.5 and E17.5 fetal mice. [file supplementary_table_2.pdf]

Supplementary Table 2. Echocardiography parameters measured in E15.5 and E17.5 fetal mice.

Pregnant dams were administered dexamethasone (Dex, 100µg/kg/day) or vehicle (Veh) in their drinking water from E12.5 to E15.5. Fetal cardiac function was assessed at E15.5 or E17.5 using a Visualsonics Vevo 770 High Resolution Ultrasound Scanner using Doppler mode. IVCT: isovolumetric contraction time, IVRT: isovolumetric relaxation time, ET: ejection time, MPI: myocardial performance index, calculated as (IVCT+IVRT)/ET, HR: heart rate measured as beats per minute (bpm), E/A: E/A wave ratio, DT: deceleration time. Data are means ± SEM and were analysed by 2-way ANOVA, with post-hoc Bonferroni tests. P values are stated where p=0.1 to 0.05. Significantly different compared to same treatment and genotype at E15.5, $p<0.05$, $p<0.001$, n=7-29 fetuses, from 5-6 litters per group.

**E15.5**							
	**Veh**		**Dex**		**ANOVA**		
**Parameter**	**Control (n=15)**	**SMGRKO (n=24)**	**Control (n=14)**	**SMGRKO (n=28)**	**Treatment**	**Genotype**	**Interaction**
IVCT (ms)	27.36 1.62	27.97 1.11	32.71 2.14	29.09 1.77	p=0.068	NS	NS
IVRT (ms)	40.92 1.48	42.66 1.33	41.43 1.15	40.67 1.09	NS	NS	NS
ET (ms)	109.44 4.44	106.60 2.41	114.08 2.86	106.22 2.65	NS	p=0.090	NS
MPI	0.64 0.04	0.66 0.02	0.65 0.03	0.66 0.03	NS	NS	NS
E/A	0.33 0.03	0.34 0.02	0.36 0.02	0.32 0.02	NS	NS	NS
DT (ms)	56.34 3.68	48.87 2.91	61.99 5.17	53.97 3.52	NS	p=0.054	NS
HR (bpm)	211.1 6.6	216.7 4.3	198.9 6.9	214.4 5.9	NS	p=0.092	NS
**E17.5**							
	**Veh**		**Dex**		**ANOVA**		
	**Control (n=18)**	**SMGRKO (n=23)**	**Control (n=8)**	**SMGRKO (n=20)**	**Treatment**	**Genotype**	**Interaction**
IVCT (ms)	29.58 1.91	31.09 2.03	26.62 1.92	29.43 1.26	NS	NS	NS
IVRT (ms)	35.59 0.98	33.90 0.85	36.77 1.06	33.94 1.06	NS	p<0.05	NS
ET (ms)	99.20 2.06	104.31 2.20	102.45 2.86	99.02 2.03	NS	NS	p=0.091
MPI	0.68 0.03	0.61 0.02	0.63 0.04	0.61 0.02	NS	NS	NS
E/A	0.47 0.03	0.46 0.02	0.45 0.02	0.42 0.02	NS	NS	NS
DT (ms)	46.35 2.31	44.28 2.37	49.35 3.15	44.85 2.94	NS	NS	NS
HR (bpm)	223.52 6.47	224.16 5.37	215.38 8.87	231.38 6.54	NS	NS	NS
