# Supplementary figures and images for: Antenatal dexamethasone treatment transiently alters diastolic function in the mouse fetal heart
Source: J Endocrinol. 2019 Apr 23;241(3):279–92. doi: 10.1530/JOE-18-0666 (PMC6541236; doi:10.1530/JOE-18-0666)

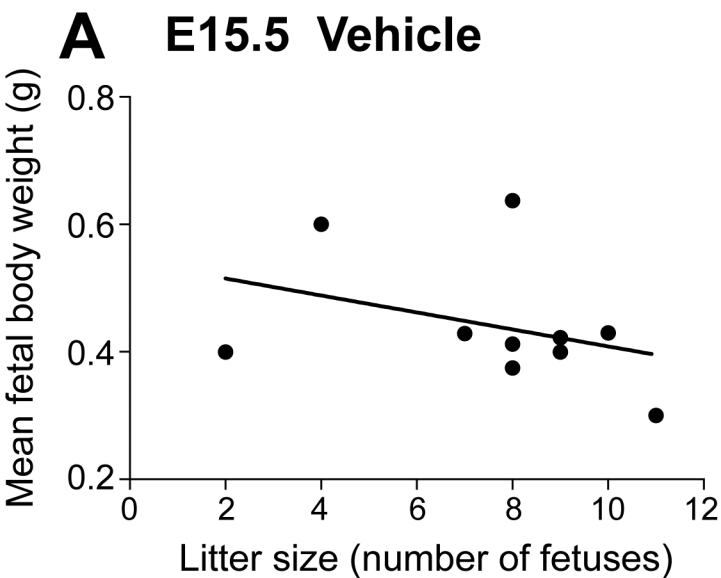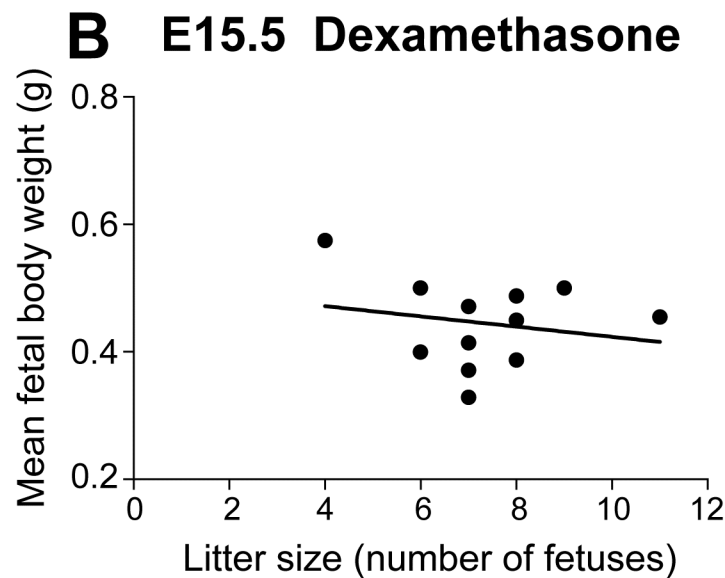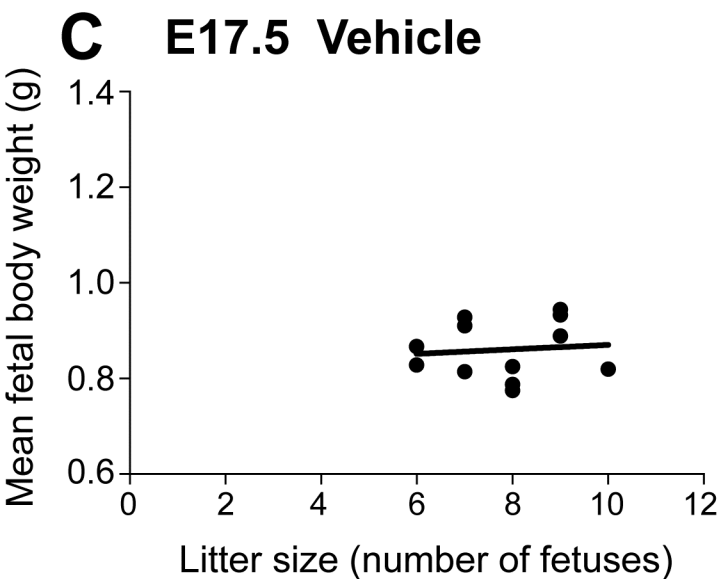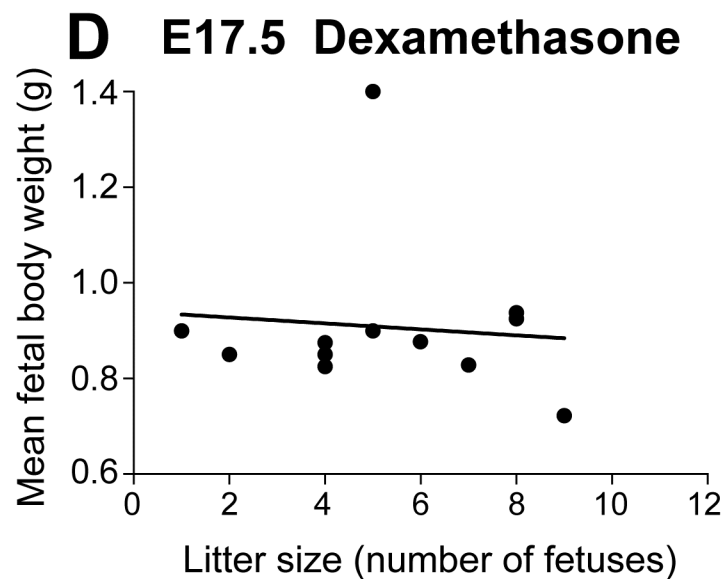

Supplement: Supplementary Figure 1. No association between mean fetal weight per litter and litter size, in vehicle and dexamethasone treated mice. Dexamethasone (Dex: 100µg/kg/day) or vehicle (Veh) was administered from E12.5 to E15.5. Individual data points represent mean fetal weight per litter (g). R2 value [file supplementary_figure_1.pdf]

## Supplementary Figure 2

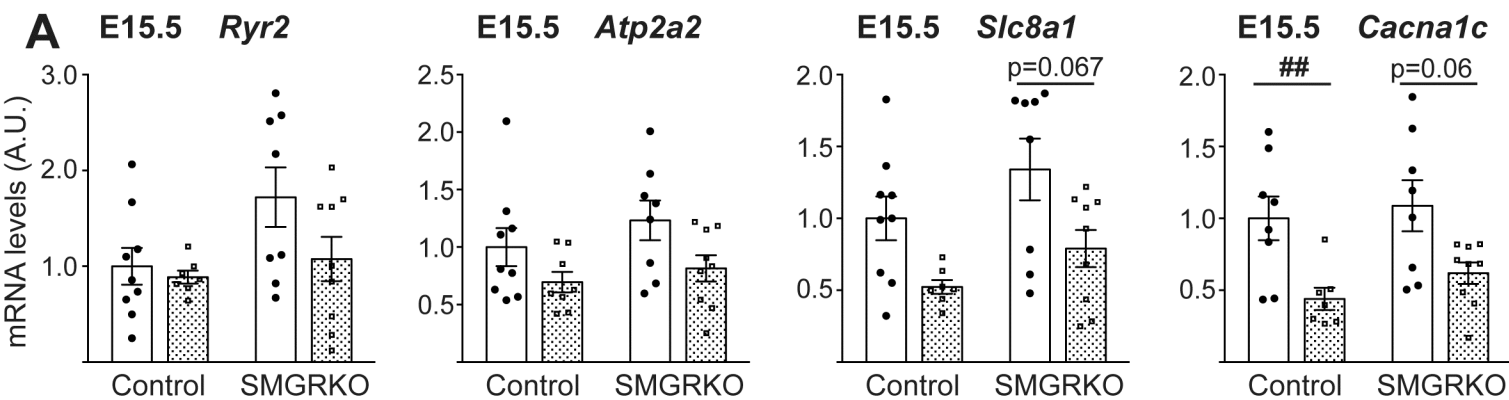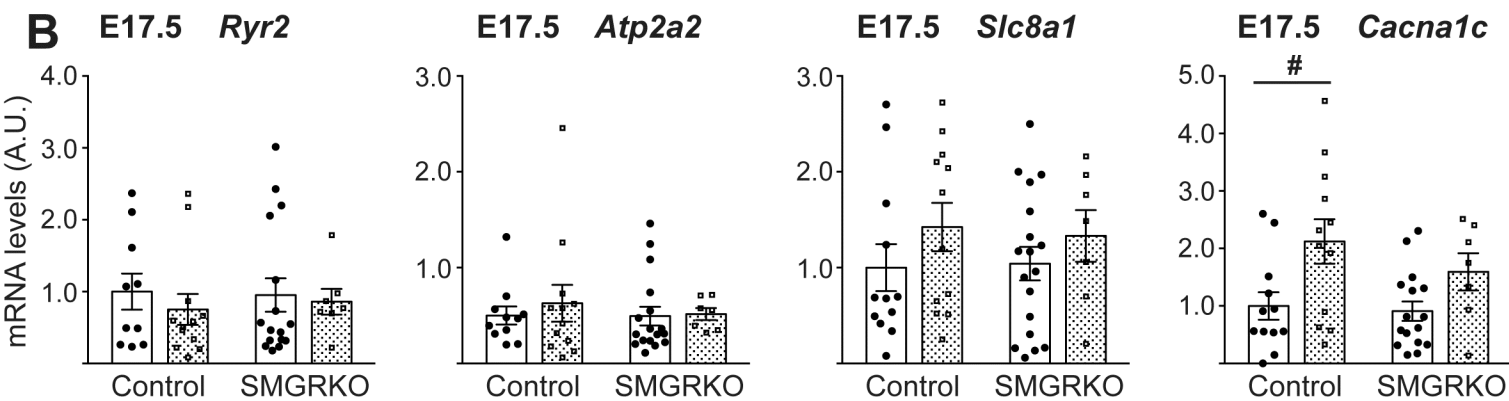

Supplement: Supplementary Figure 2. Levels of mRNA encoding calcium handing proteins are altered at E15.5 by dexamethasone treatment. Dexamethasone (100µg/kg/day, stippled bars) or vehicle (Veh, white bars) was administered to pregnant dams in their drinking water from E12.5 to E15.5. qRT-PCR measurements at (A [file supplementary_figure_2.pdf]

Supplementary Figure 3

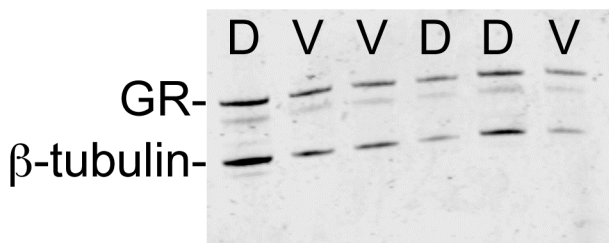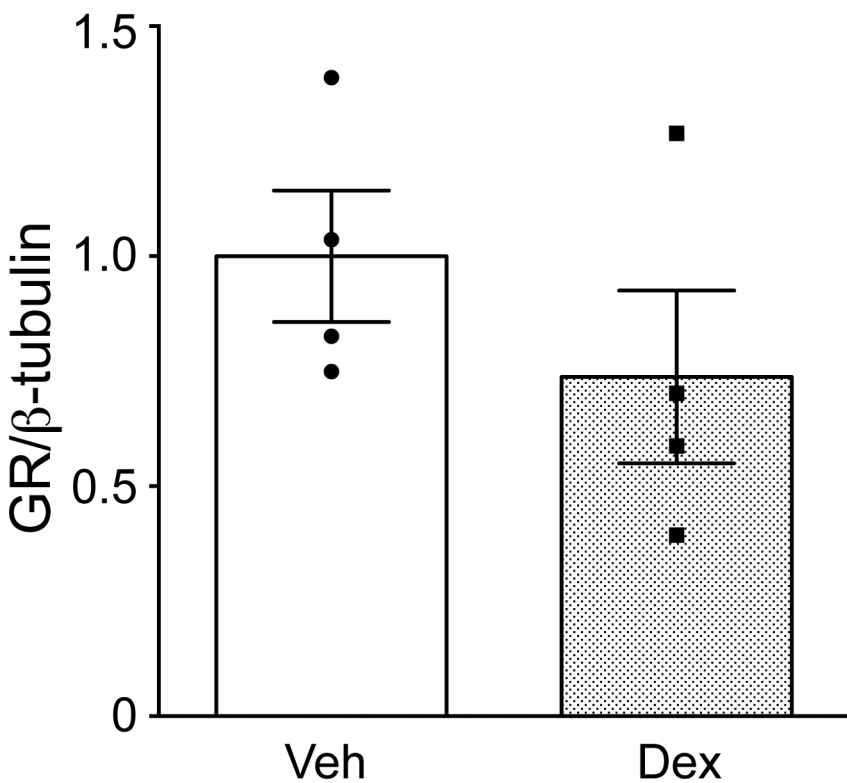

Supplement: Supplementary Figure 3. GR protein levels in control fetal hearts at E15.5. Western blotting (representative image in upper panel) was used to measure protein levels in hearts of vehicle (V/Veh) and dexamethasone (D/Dex) treated mice. Graph shows quantification of GR protein levels relative to β-tub [file supplementary_figure_3.pdf]

# Supplementary Figure 4

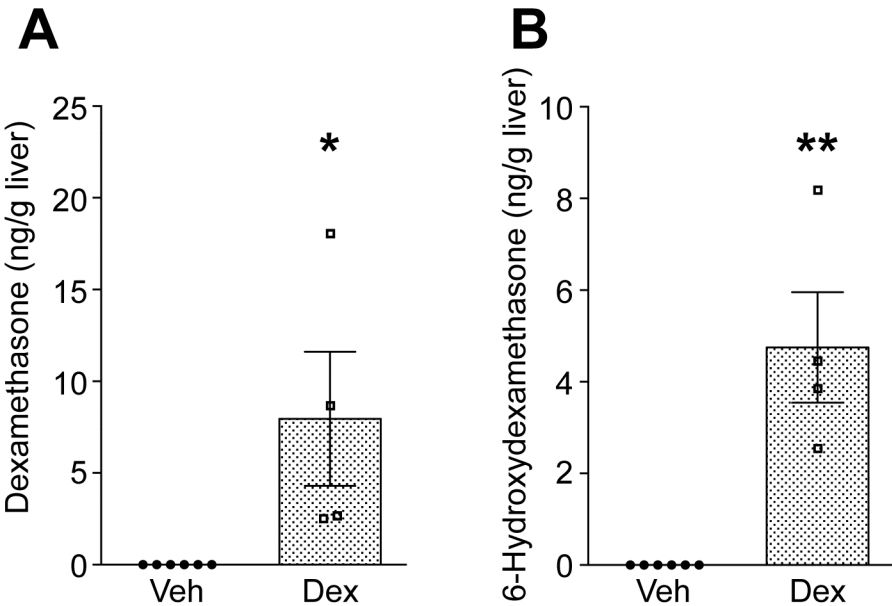

Supplement: Supplementary Figure 4. Dexamethasone and its 6-hydroxydexamethasone metabolite are present in livers of dexamethasone-treated dams at E15.5. Dexamethasone (Dex: 100µg/kg/day) or vehicle (Veh) was administered from E12.5 to E15.5. Steroids were measured in maternal livers by mass spectrometry. The d [file supplementary_figure_4.pdf]

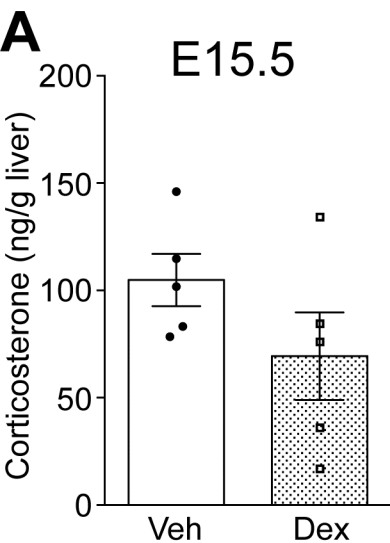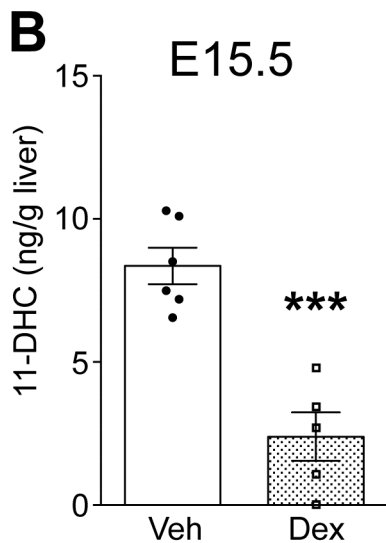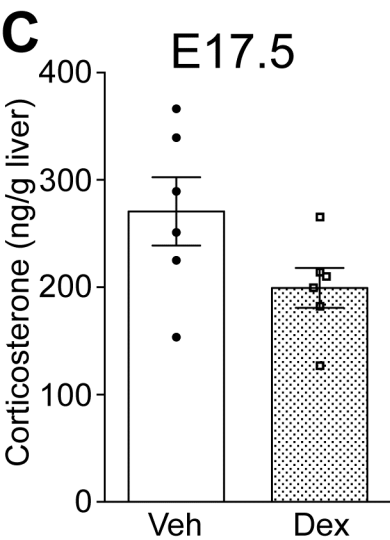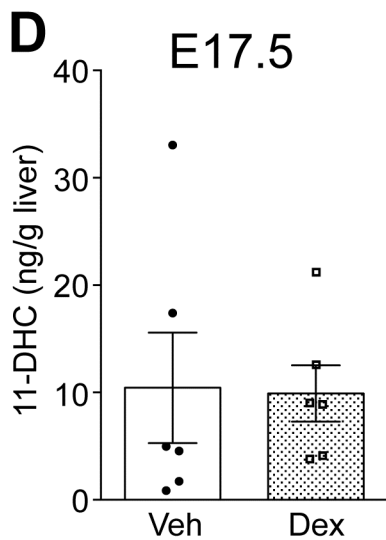

Supplement: Supplementary Figure 5. Endogenous glucocorticoid levels in dam liver at E15.5 and E17.5. Dexamethasone (Dex: 100µg/kg/day) or vehicle (Veh) was administered from E12.5 to E15.5. Steroid levels were measured in maternal livers by mass spectrometry: (A) corticosterone at E15.5, (B) 11-dehydrocorticos [file supplementary_figure_5.pdf]

# Supplementary Figure 6

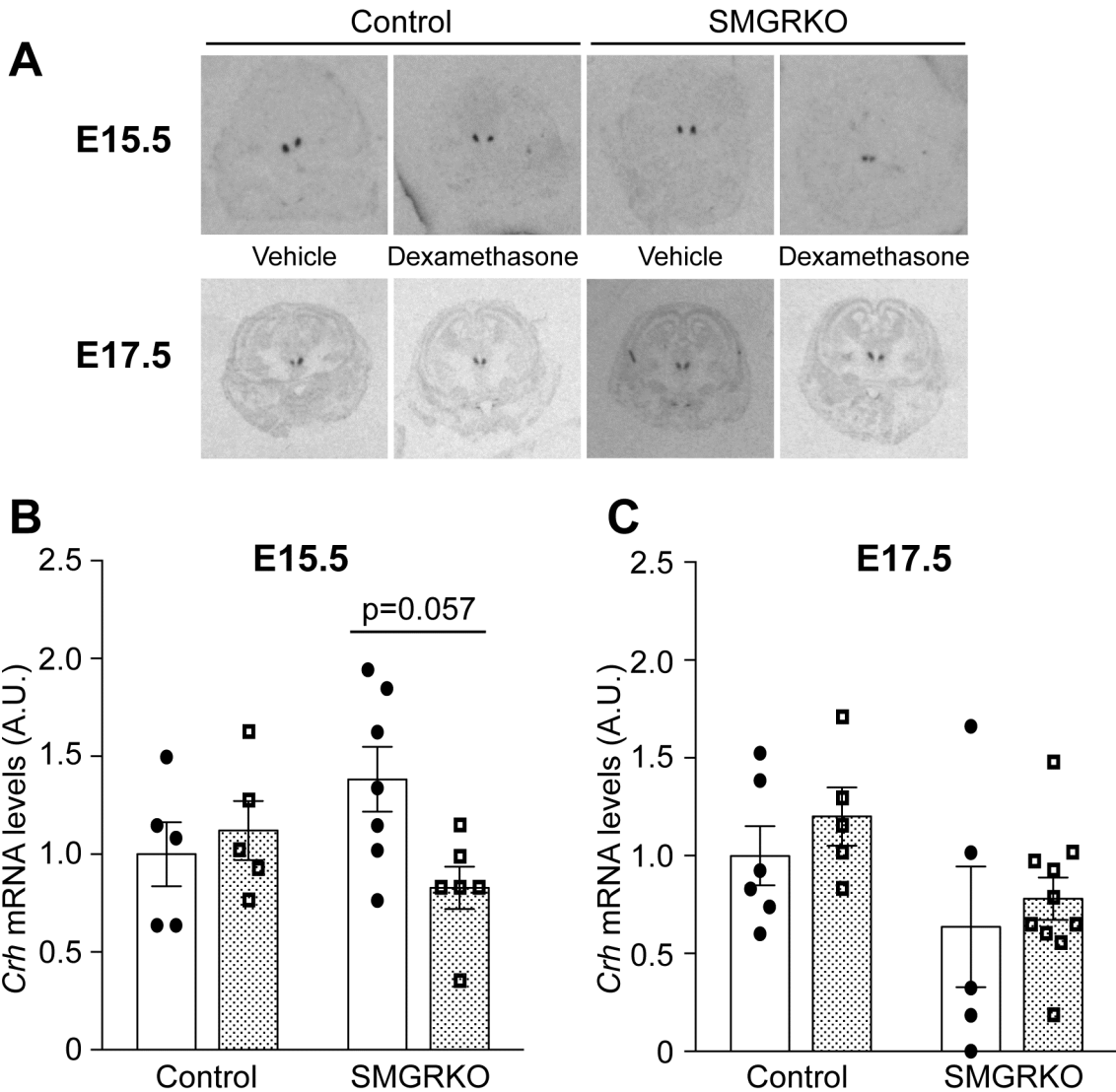

Supplement: Supplementary Figure 6. SMGRKO fetal HPA axis is downregulated at E15.5 following dex exposure, with no change at E17.5 or in the control ‘floxed’ group. Corticotropin releasing hormone (Crh) mRNA levels were measured bilaterally in the PVN of the hypothalamus by in situ hybridisation. (A) Represent [file supplementary_figure_6.pdf]
